# The impact of peer mentoring on leadership and self-efficacy in higher music education: a mixed-methods study

**DOI:** 10.3389/fpsyg.2026.1756556

**Published:** 2026-02-09

**Authors:** Zaihao Wu, Cheng Yao, Na Yang

**Affiliations:** 1 School of Music and Dance, Xihua University, Chengdu, China; 2 Faculty of Education, Nakhon Phanom University, Nakhon Phanom, Thailand; 3 Academic Affairs Office, Lijiang Normal University, Lijiang, China

**Keywords:** conservatoire training, higher music education, leadership development, musical self-efficacy, peer learning, peer mentoring

## Abstract

**Introduction:**

Peer mentoring programs (PMPs) are increasingly used in higher education, yet evidence of their dual effects on mentors’ leadership and trainees’ musical self-efficacy remains limited in higher music education, particularly in non-Western conservatoire contexts.

**Methods:**

An explanatory sequential mixed-methods design was employed. Quantitative data were collected from 32 peer mentor–peer trainee pairs (*N* = 64) participating in a two-month mentoring program, with leadership and musical self-efficacy measured at pretest, posttest, and one-month follow-up. Within-group changes were analyzed using repeated-measures ANOVA. Semi-structured interviews with 20 participants were conducted to further explore mentoring experiences and underlying mechanisms.

**Results:**

Peer mentors demonstrated significant and sustained improvements in leadership behaviors, whereas leadership change among trainees was limited. In contrast, peer trainees showed substantial and sustained gains in musical self-efficacy, while mentors’ self-efficacy exhibited only modest change. Qualitative findings indicated that mentors developed greater organizational awareness and role responsibility, while trainees benefited from emotional support, targeted feedback, and vicarious learning.

**Conclusion:**

This study demonstrates that peer mentoring programs can be effectively integrated into higher music education. Participation in structured peer mentoring was associated with leadership development among mentors and improved musical self-efficacy among trainees, highlighting the value of role-based peer interaction as a complementary approach to traditional conservatoire pedagogy.

## Background

Peer mentoring is often traced to Athena’s guidance of Telemachus in Greek mythology, a symbolic precursor to the idea of guided support. In modern education, it refers to a structured relationship in which experienced students provide academic, emotional, and social support to peers with less experience (Krisi and Nagar, 2023). In contemporary higher education, Peer Mentoring Programmes (PMPs) are widely implemented to support students’ transition into university life. Research indicates that PMPs’ participation reduces anxiety, enhances learning motivation, improves social integration, and fosters a safe, trustworthy, and supportive learning environment beyond formal classrooms (Kachaturoff et al., 2020; Liu et al., 2022). Within the role structures of PMPs, students with relevant experience are identified as Peer Mentors (PMs), while those in the early stages of learning who require support are designated as Peer Trainees (PTs) (Vulliamy and Junaid, 2013; Williams et al., 2020).

However, the mechanisms through which PMPs operate vary across disciplines and exhibit distinctive characteristics in the highly specialized field of music education. Gaunt and Westerlund (2013) note that higher music education has traditionally relied on a master–apprentice pedagogy, with limited attention to social interaction, collaborative learning, and peer learning, despite evidence that deeper learning emerges through ensemble rehearsal, collaborative practice, and reflective dialogue. Similarly, Hanken (2016) argues that conservatoires’ emphasis on one-to-one instruction can limit students’ engagement in each other’s learning, highlighting the need to strengthen peer learning within principal instrument study and teaching practice. Reviewing 51 studies published between 1980 and 2021, Fernández-Barros et al. (2022) conclude that peer tutoring, as a form of cooperative learning, holds substantial potential in both formal and informal music education and can reduce learning barriers. Goodrich (2007, 2021a, 2021b) further demonstrates that across jazz ensembles, extracurricular instruction, and online music learning, peer mentoring fosters interaction, supports meaning-making, and improves rehearsal and instructional efficiency. However, existing research in music education has largely focused on learning outcomes and pedagogy, with limited attention to mentors’ leadership development (Ilchuk et al., 2024).

PMPs provide not only emotional and academic support but also align closely with the cyclical processes of modeling, feedback, practice, and reflection that underpin musical skill development in music learning (Goodrich et al., 2018). Musical learning depends heavily on observing demonstrations, receiving targeted feedback, and making continuous adjustments during practice, making peer interaction an essential learning resource. Less experienced music performance students often face uncertainty in technique, practice strategies, and stage performance, which can heighten self-doubt and performance anxiety and, in turn, undermine learning motivation and professional identity development (Barros et al., 2022). In this context, PMPs offer PTs vicarious learning opportunities, timely feedback, and emotional support, helping them better grasp learning strategies, reduce anxiety, and enhance self-efficacy and engagement (Jones and Wendt, 2025).

In research on music learning and peer support, self-efficacy refers to students’ beliefs in their capability to organize and execute the actions required to successfully perform learning tasks and cope with academic challenges. This understanding draws on Bandura’s (1977) social cognitive theory, which emphasizes that self-efficacy is shaped through experience and varies across contexts. In music learning, self-efficacy is closely related to students’ confidence in technical execution, practice strategies, and performance preparation, and has been shown to influence persistence, learning engagement, and performance-related anxiety (Ou and Qin, 2025; Ou et al., 2025).

The developmental value of PMPs for PMs has increasingly attracted scholarly attention. Research indicates that students who assume mentoring responsibilities demonstrate improvements in organisational coordination, communication, initiative, and self-regulation, all of which contribute to leadership development (Murrell et al., 2021). Leadership is conceptualized as an identity that develops gradually through participation in social tasks. When supported by clear role expectations, authentic practice opportunities, and ongoing feedback, PMs are more likely to internalize a leadership identity and enhance their capacity for effective action (Lowery et al., 2019). Moreover, leadership development in peer mentoring is shaped by role and relational dynamics and is neither automatic nor uniform (Cortellazzo et al., 2023).

Although the effectiveness of PMPs has been well documented in fields such as engineering and medicine (Ballesteros et al., 2024; Preovolos et al., 2024), research within Chinese higher music education remains limited. These gaps are especially salient in non-Western, performance-oriented contexts shaped by distinct pedagogical traditions and learning cultures (Rector, 2025). Traditional music instruction is predominantly teacher-centred and provides limited opportunities for peer collaboration or support. Existing studies have focused primarily on PMPs’ effects on PTs, while their dual influence on PMs has received comparatively little attention. To address this gap, the present study implemented two-month PMPs within the music performance programme of a Chinese university. The programme employed a clearly defined PM–PT role structure to investigate the mechanisms through which peer mentoring shapes leadership development in PMs and enhances self-efficacy in PTs.

The primary research question of this study are:

(1) How can PMPs be designed and implemented to align with the characteristics of higher music education?
(2) How does participation in PMPs influence leadership development among PMs?
(3) How does participation in PMPs influence self-efficacy among PTs?

## Method

### Research design

The study employed an explanatory sequential mixed-methods design (Creswell and Plano Clark, 2018) to examine the effects of Peer Mentoring Programmes (PMPs) on students’ leadership behaviors and musical self-efficacy. In the quantitative phase, a quasi-experimental pretest–posttest design was used to assess music performance majors who participated in a two-month intervention. Second-year students served as PMs (Group A), while first-year students served as PTs (Group B). Both groups completed a pretest, posttest, and a one-month follow-up assessment to examine change patterns and short-term maintenance of intervention effects. A one-month follow-up assessment was selected because this time frame is widely used in related research, offers strong practical feasibility, and helps minimize participant attrition as well as disruption to the academic schedule (eg. Cai et al., 2008). Given inherent differences in grade level, role expectations, and learning tasks, no between-group comparisons were conducted. Instead, the analysis focused on within-group changes over time to inform the development of subsequent interview questions. Participation in the PMPs served as the independent variable, whereas leadership behaviors and musical self-efficacy were the dependent variables.

In the qualitative phase, semi-structured interviews were conducted to further explore students’ experiences, including role interactions, learning strategies, emotional support, and collaborative practices. Qualitative data were used to interpret quantitative trends, capture process-oriented experiences beyond the reach of quantitative measures, and trace students’ participation pathways during the intervention, thereby deepening understanding of the mechanisms underlying capability development.

**Figure 1:**
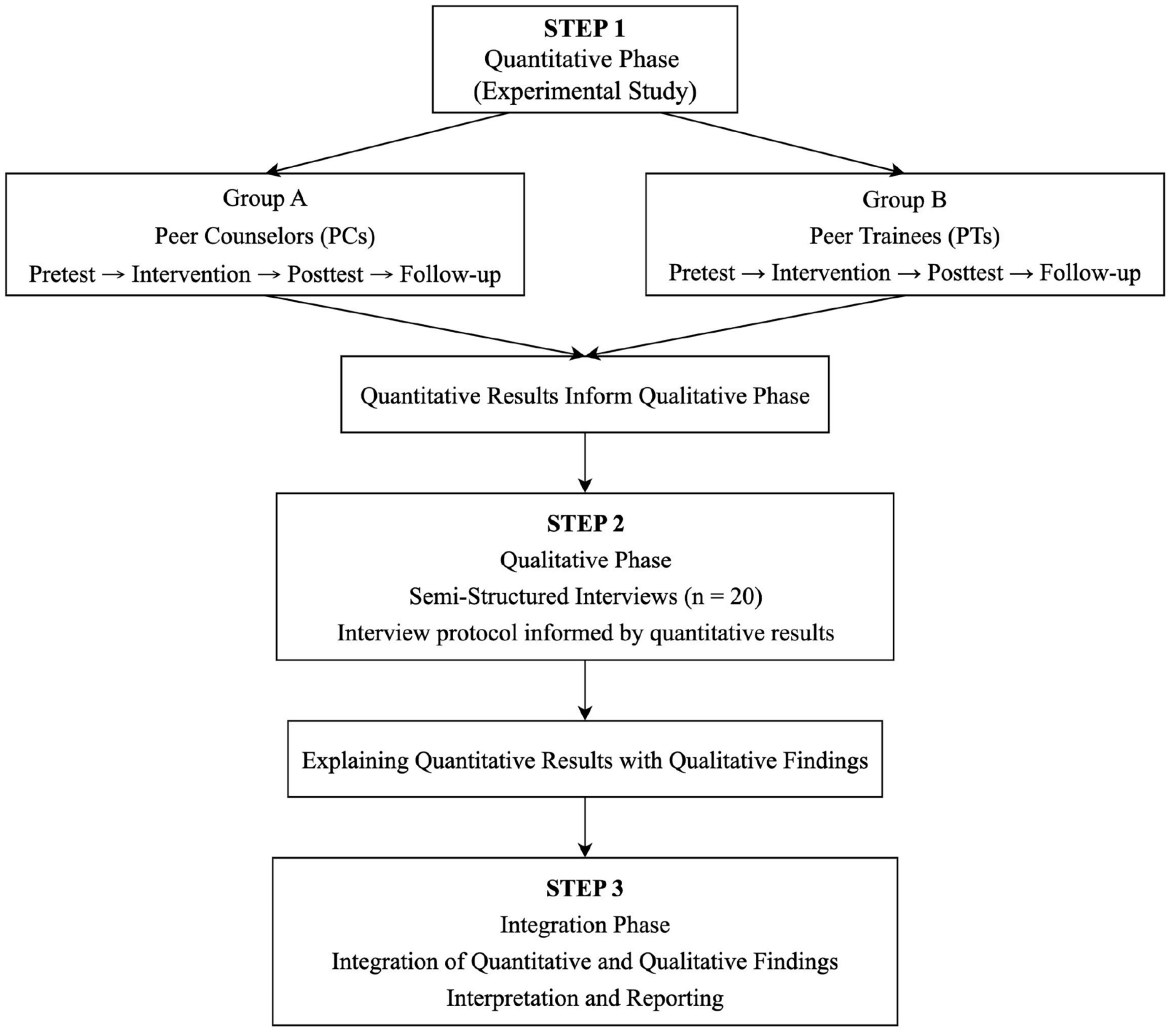
Procedural overview of the explanatory sequential mixed-methods study. PCs = peer counselors; PTs = peer trainees. The study used an explanatory sequential mixed-methods design, with quantitative results informing the qualitative phase and qualitative findings explaining the quantitative results. Integration occurred during interpretation and reporting.

During the integration phase, the study examined convergence and complementarity between quantitative change patterns and qualitative themes. By synthesizing outcome-level findings with process-level experiences, a holistic understanding was developed of how PMPs operate and their broader educational significance. Figure 1 illustrates the procedural overview of the explanatory sequential mixed-methods study.

### Participants

The study was conducted at the School of Music of X University in China during the 2024–2025 academic year. A total of 255 undergraduate students majoring in Music and Performance constituted the eligible population for the study. Second-year students were assigned as PMs, and first-year students as PTs. PMs were selected through faculty nomination and research-team screening based on professional competence, academic performance, communication skills, and responsibility. The mentoring role was voluntary and independent of degree requirements, and all PMs received full information and training before participation. PTs joined through voluntary registration according to their learning needs and were subsequently matched within the programme.

To ensure rigorous one-to-one matching, the research team collected baseline demographic information (e.g., gender, age, musical family background, and prior tutoring experience) as well as participants’ specialization, skill focus, learning goals, and availability. Potential pairings were rated on a 0–10 scale based on specialization, skill characteristics, academic level, learning interests, and scheduling compatibility, with scores ≥7 indicating high-priority matches, 5–6 moderate matches, and ≤4 requiring reassignment. Two research assistants independently conducted the ratings and resolved discrepancies through discussion. Pairings were fixed once established to ensure intervention continuity. Detailed procedures are provided in Appendix A. For example, a second-year piano major with strong academic performance closely aligned with a first-year piano major could receive a matching score of 9 and be prioritized for pairing.

When group sizes differed, pairings followed the principle of similarity. Students were excluded if they lacked an available partner, failed the training assessment, had incomplete data, did not complete the intervention, or were absent for more than two weeks or withdrew. Ultimately, 32 PM–PT pairs (64 participants) were formed for the quantitative analysis. This sample size reflects the practical constraints of a longitudinal, one-to-one mentoring design, including sustained participation, fixed dyadic matching, and repeated measurements, and is comparable to that reported in prior PMPs (Smith et al., 2017). For the qualitative phase, based on data saturation principles (Wutich et al., 2024), ten PMs and ten PTs were subsequently selected for semi-structured interviews. PMs received a 100-yuan supermarket voucher, and PTs a commemorative notebook as compensation.

### Development of the peer mentoring programs

The PMPs in this study were grounded in Social Learning Theory (Bandura, 1977), Scaffolding Theory (Wood et al., 1976), and prior research on collaborative learning in music education. Following an initial needs analysis, the research team developed an initial draft and invited five experts in music education and performance psychology to assess its content validity using the Item–Objective Congruence index. The experts rated each activity on a three-point scale (1 = congruent, 0 = uncertain, −1 = incongruent) and offered feedback for refinement. Based on the expert evaluations, the research team completed three rounds of revision. After refinement, all items obtained mean Item–Objective Congruence index scores ranging from 0.80 to 1.00 (see Appendix B), demonstrating strong content validity (Turner and Carlson, 2003). The final PMPs comprised four functional modules (see Table 1): emotional support, skill-based assistance, experience sharing, and stage preparation.

**Table 1:** Peer mentoring programs: four-module instructional flowchart.

Characteristic	Emotional support	Skill enhancement	Experience sharing	Stage preparation
Objective	Establish trust, alleviate anxiety	Enhance accuracy, rhythmic control, detail	Equip with practice strategies, routines	Develop stage adaptability, readiness
Activity 1	Icebreaker dialogues, Introductions	Segmented rehearsal led by mentors	Experience talk sessions	Repertoire coaching
Activity 2	Emotion mapping and sharing	Technique deconstruction, demonstration	Scenario-based simulation	Stage movement and expressive training
Activity 3	Mentors’ personal narratives	Weekly video feedback sessions	Interactive Q&A	Performance readiness assessment

To ensure PMs competency, two doctoral-level instructors with more than five years of professional experience delivered Peer Mentoring Training that covered communication skills, musical guidance strategies, feedback techniques, and collaborative dispositions. Participation in the PMPs was not tied to degree requirements. PMs were required to complete one week of training (approximately 10 h) and pass an assessment before entering the intervention phase, a duration that balanced adequate preparation with academic feasibility.

Before formal mentoring began, each PM–PT dyad discussed collaborative goals, communication methods, boundaries, and expectations to clarify the distinction between the roles of PMs and professional instructors. The mode of mentoring (face-to-face, online, or hybrid) was jointly determined by both parties. Over the two-month intervention, each dyad conducted at least eight mentoring sessions of no less than 40 min each, ensuring that all four modules were practiced at least twice. Additional interaction was allowed when needed. Throughout the intervention, the research team provided ongoing support and collected student feedback at completion to inform continuous program refinement.

### Data collection

#### Quantitative phase: experimental research

The quantitative phase employed a pretest–posttest experimental design implemented over a two-month intervention (July–September 2025). The intervention centred on structured tasks supplemented with student-initiated informal interactions. All PM–PT dyads completed the four formal mentoring modules as scheduled and engaged in additional learning exchanges as needed, and official activities were documented through weekly logs.

Both Group A and B used the same assessment instruments across all measurement sessions to ensure consistency. One week before the intervention, all participants completed two baseline measures: the Student Leadership Behavior Scale (SLBS) and the General Musical Self-Efficacy Scale (GMSES). The SLBS consists of 30 items across six dimensions rated on a 5-point Likert scale and has demonstrated strong internal consistency and structural validity in prior research (Kimura et al., 2022) (see Appendix C). The GMSES includes two subscales comprising 22 items rated on a 7-point Likert scale, with well-established reliability and convergent validity among music learners (Casanova et al., 2023) (see Appendix D).

To ensure applicability for Chinese music majors, the study conducted cross-cultural translation and localization of both instruments. Two bilingual researchers conducted forward translation and back-translation, followed by expert evaluation from three domain specialists who assessed linguistic accuracy and cultural relevance. Revisions were made based on expert feedback, and the instruments were subsequently piloted with 35 students before final adjustments. The internal consistency coefficients in the main sample were satisfactory (SLBS: *α* = 0.814; GMSES: α = 0.833), indicating that both instruments were appropriate for the study context.

At the end of the intervention, all participants completed a posttest identical to the pretest to assess changes over time. A follow-up test was administered one month later using the same measures to evaluate the durability of the intervention effects.

#### Qualitative phase: semi-structured interviews

To complement the quantitative data, semi-structured interviews were conducted one week after the follow-up assessment. Using purposive sampling, the study selected “information-rich cases” capable of providing key insights (Ahmad and Wilkins, 2025). To avoid an overly concentrated sample, maximum variation sampling was additionally employed to ensure heterogeneity across gender, specialization, pairing compatibility level (high, medium, low), degree of participation, and interaction patterns, thereby capturing a diverse range of perspectives (Staller, 2021).

The interview sample size was determined by qualitative data saturation. Recruitment and analysis were conducted concurrently and continued until no substantively new themes emerged (Wutich et al., 2024). A total of 20 students were ultimately interviewed, including 10 peer mentors (PMs, C1–C10) and 10 peer trainees (PTs, T1–T10). Participants were not automatically recruited as mentor–mentee dyads, and both partners were included only when each was able to provide information-rich perspectives. The remaining interviewees were selected independently using maximum variation sampling to ensure diversity and reduce homogeneity bias.

The interview protocol was developed based on the literature review, research objectives, and preliminary quantitative findings (see Table 2). To ensure alignment between each question and the research aims, five experts also evaluated the content validity of the interview guide and provided suggestions regarding wording and structure. Revisions were made accordingly, and all items achieved mean Item–Objective Congruence values of 0.80 or higher, indicating strong content validity (Appendix E).

**Table 2:** Interview outline.

Role	Topic	Interview questions
PMs (Group A)	Guidance experiences and challenges	Q1: What was the biggest challenge you encountered during your time as a peer mentor?
	Changes in responsibility	Q2: Has being a peer mentor improved your organisational and leadership skills?
	Professional growth	Q3: Has mentoring others helped you deepen your understanding of music or career development?
PTs (Group B)	Experience	Q1: What was the most rewarding experience you had when receiving help from a peer mentor?
	Changes in learning confidence	Q2: Has this programme boosted your confidence in your musical studies?
	Future expectations	Q3: Would you like to be a peer mentor in the future?

Prior to the interviews, researchers explained the study purpose, confidentiality principles, and use of audio recordings, and obtained written informed consent from all participants. Each interview was conducted by two researchers. One researcher led the questioning, while the other handled note-taking and equipment management. All interview sessions were audio-recorded. Interviews lasted approximately 20–30 min and took place in private meeting rooms or other quiet locations. The interview procedure followed five steps: scheduling and confirmation, introductory briefing, transitional questions, core questions, and closing remarks, thereby ensuring a clear structure and natural interaction. All recordings were transcribed verbatim for subsequent thematic analysis.

### Data analysis

This study adopted an explanatory sequential mixed-methods design, with data analysis comprising quantitative and qualitative components, followed by an integration phase in which findings were compared and synthesized to achieve complementarity.

Quantitative analyses were conducted using SPSS 28.0. Descriptive statistics were first calculated for leadership and self-efficacy variables, including means, standard deviations, and 95% confidence intervals. Repeated-measures ANOVA was then employed to examine within-group changes across three time points (pretest, posttest, and follow-up) for Groups A and B. Consistent with the quasi-experimental design, this analysis was used to identify temporal change patterns within each group rather than to test causal or intervention effects. Accordingly, the results were interpreted as developmental trends associated with participation in the peer mentoring programme, and no between-group comparisons or causal inferences were made. When Mauchly’s test indicated a violation of the sphericity assumption, the Greenhouse–Geisser correction was applied. Paired-sample t-tests were conducted, where appropriate, to clarify the direction of within-group differences. Partial η^2^ was reported as the effect size, together with interval estimates for estimated marginal means. Internal consistency reliability for both measurement scales was also examined within the study sample. Participants with incomplete data were excluded prior to analysis, thus, all quantitative analyses were based on complete datasets.

The qualitative component employed Braun and Clarke’s (2006, 2019) inductive semantic thematic analysis. All verbatim transcripts were imported into NVivo 12.2 to support data management and coding. Researchers first conducted open coding of the verbatim transcripts to identify recurring keywords or concepts, which were subsequently clustered into higher-order themes. To ensure credibility, two researchers resolved discrepancies through discussion, and when necessary, a third scholar with qualitative research expertise was consulted for peer debriefing, thereby enhancing coding consistency and overall analytical rigor. All interviews were completed in full, with no substantive qualitative data missing. The finalized themes captured key experiences of PMs and PTs and were supported with representative quotations.

During the integration phase, qualitative themes were used to interpret and extend the quantitative findings. They helped explain why certain dimensions showed either significant or limited change and illuminated process-level experiences that quantitative methods could not capture, such as interaction quality, emotional support, and strategy use. Together, these integrated insights offered a more comprehensive understanding of the mechanisms and effects of the PMPs (Creswell and Plano Clark, 2018).

### Ethical approval

The studies involving human participants were reviewed and approved by Xihua University (Ref. No. XH20250104-04) and were conducted in accordance with local legislation and institutional requirements. All participants provided written informed consent prior to participation.

## Results

### Quantitative results

#### General characteristics

Table 3 shows that both groups were relatively balanced in gender distribution, with slightly more females than males. Group A had a higher average age (19.8) than Group B (18.7). The proportion of students with a family music background or extracurricular tutoring was comparable across groups. These similarities in baseline characteristics suggest that the two groups were demographically aligned.

**Table 3:** Participant characteristics.

Variable	Category	A Group (*n* = 32)	B Group (*n* = 32)
Gender	Male	13 (40.6%)	14 (43.8%)
	Female	19 (59.4%)	18 (56.2%)
Age (years)	Mean ± SD	19.8 ± 0.6	18.7 ± 0.5
Family with music background	Yes	11 (34.4%)	10 (31.2%)
	No	21 (65.6%)	22 (68.8%)
Received extracurricular tutoring	Yes	9 (28.1%)	12 (37.5%)
	No	23 (71.9%)	20 (62.5%)
Group role	Role assigned	Peer mentors	Peer trainee

#### Leadership

Prior to the repeated-measures ANOVA, data normality was assessed using the Shapiro–Wilk test. Results indicated no substantial departures from normality across measurement occasions (all *p* > 0.05), supporting the use of parametric analyses.

Group A demonstrated a marked improvement in leadership while serving as PMs. The repeated-measures ANOVA revealed a strong and statistically significant main effect of time, *F* (2, 62) = 143.49, *p* < 0.001, and the sphericity assumption was satisfied (W = 0.928, *p* = 0.325). The mean score increased from 113.69 at pretest to 117.53 at posttest and stabilized at 116.38 during follow-up, with corresponding 95% confidence intervals of [111.81, 115.56], [115.58, 119.48], and [114.48, 118.27]. All pairwise comparisons were statistically significant (*p* < 0.001). The effect size further indicated a robust influence of time on leadership performance (partial η^2^ = 0.82). Overall, leadership scores in Group A showed a substantial increase over time, which was maintained at follow-up.

In contrast, Group B exhibited minimal change in leadership across the intervention period. Because the sphericity assumption was violated (W = 0.610, *p* < 0.001), the Greenhouse–Geisser correction was applied. After correction, the main effect of time was no longer significant, *F* (1.44, 44.60) = 3.50, *p* = 0.053, and the effect size remained small (partial η^2^ = 0.10). The mean scores at pretest, posttest, and follow-up (114.38, 114.50, and 114.41) showed negligible variation. Of the pairwise comparisons, only the pretest–posttest contrast reached significance (*p* = 0.044), and all others were nonsignificant. Taken together, these findings indicate that Group B maintained a stable level of leadership throughout the intervention, with no evidence of substantive improvement.

#### Self-efficacy

Group A exhibited minimal changes in self-efficacy. Although the preliminary analysis indicated a significant main effect of time, *F* (2, 62) = 4.18, *p* = 0.020, the sphericity assumption was violated (W = 0.782, *p* = 0.025). Therefore, the interpretation relied on the Greenhouse–Geisser–adjusted estimates. After correction, the time effect was marginally significant, *F* (1.64, 50.91) = 4.18, *p* = 0.028, with a partial η^2^ of 0.12, reflecting a small-to-medium effect. Mean scores increased only slightly, from 100.66 at pretest to 100.72 at posttest, and further to 100.81 at follow-up. The corresponding 95% confidence intervals were [97.59, 103.72], [97.67, 103.77], and [97.77, 103.85], respectively. Only the contrast between pretest and follow-up reached significance (*p* = 0.044). Overall, Group A demonstrated a slight upward trend in self-efficacy, but the magnitude of improvement remained limited.

Group B, in contrast, exhibited a substantial and sustained increase in self-efficacy across the intervention period. Because the sphericity assumption was violated (W = 0.596, *p* < 0.001), the Greenhouse–Geisser correction was implemented. Both unadjusted and adjusted analyses produced a highly significant main effect of time (*F* = 66.17, *p* < 0.001), accompanied by a large effect size (partial η^2^ = 0.68). Estimated marginal means increased markedly from 93.69 at pretest to 96.09 at posttest and were largely maintained at 95.81 at follow-up, with corresponding confidence intervals of [90.81, 96.56], [93.21, 98.97], and [92.89, 98.74]. Pairwise comparisons indicated significant differences between pretest and posttest and between pretest and follow-up (both *p* < 0.001), whereas the posttest–follow-up contrast did not reach significance (*p* = 0.071). These findings indicate a robust and sustained increase in self-efficacy over time within Group B.

**Figure 2:**
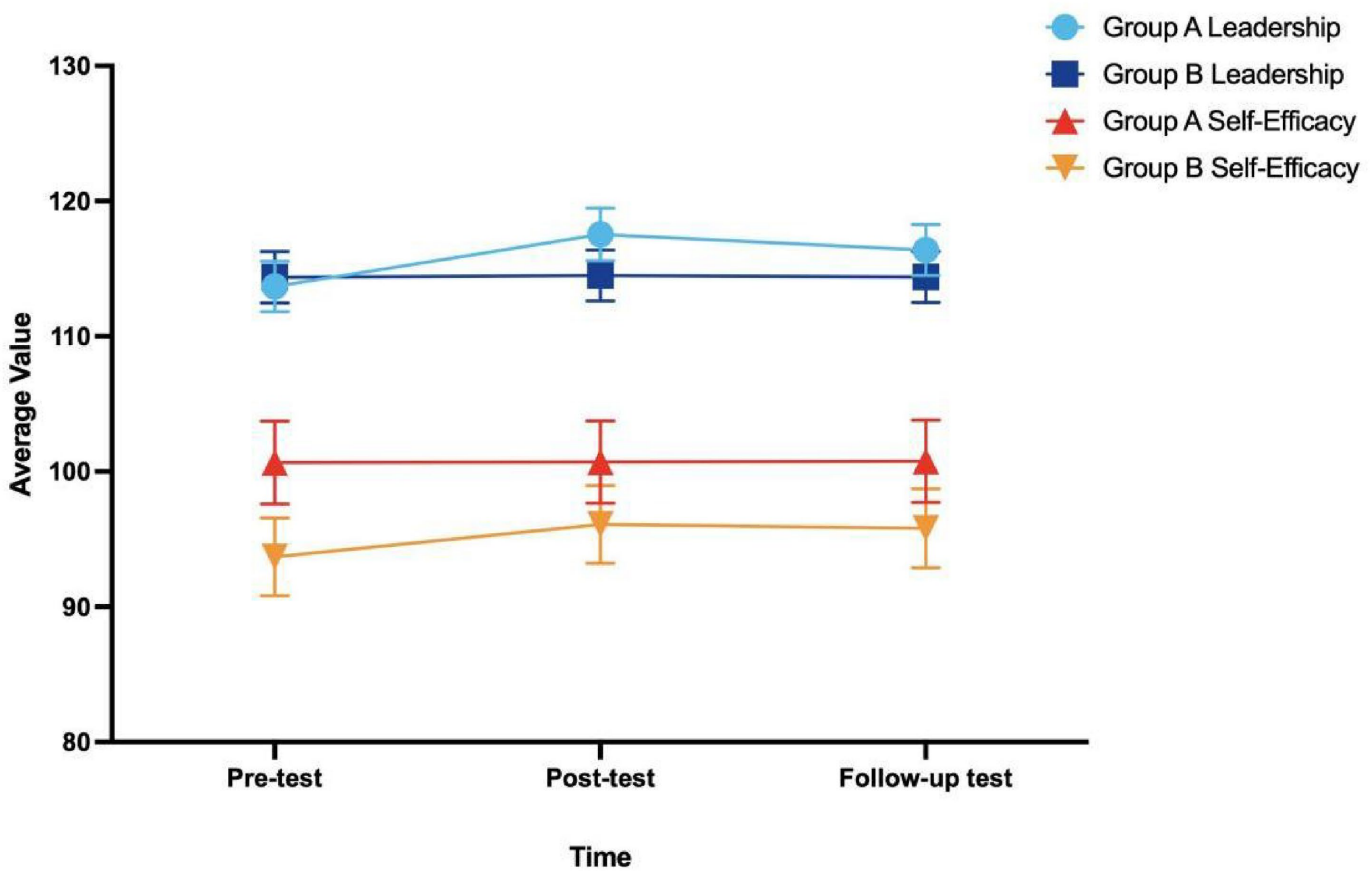
Changes in leadership and self-efficacy scores over time for Groups A and B. Values represent means, and error bars indicate 95% confidence intervals. Group A and Group B correspond to the experimental groups described in the text.

Descriptive statistics for both leadership and self-efficacy across all time points are presented in Table 4, the corresponding repeated-measures ANOVA results for each outcome are provided in Table 5, and the overall trajectories of change are visualized in Figure 2.

**Table 4:** Descriptive statistics for Group A and Group B at pretest, posttest, and follow-up.

Variable	Group	Time point	Mean	SD	95% CI
Leadership	Group A	Pretest	113.69	5.2	[111.81, 115.56]
		Posttest	117.53	5.4	[115.58, 119.48]
		Follow-up	116.38	5.26	[114.48, 118.27]
	Group B	Pretest	114.38	5.29	[112.47, 116.28]
		Posttest	114.5	5.24	[112.61, 116.39]
		Follow-up	114.41	5.26	[112.51, 116.30]
Self-Efficacy	Group A	Pretest	100.66	5.2	[97.59, 103.72]
		Posttest	100.72	5.14	[97.67, 103.77]
		Follow-up	100.81	5.18	[97.77, 103.85]
	Group B	Pretest	93.69	5.22	[90.81, 96.56]
		Posttest	96.09	5.13	[93.21, 98.97]
		Follow-up	95.81	5.22	[92.89, 98.74]

**Table 5:** Repeated-measures ANOVA results for leadership and self-efficacy in Group A and Group B.

Variable	Group	RM-ANOVA (Adjusted)	df	Mauchly’s W	Posttest – pretest	Follow-up – pretest	Follow-up – posttest	Partial η^2^
Leadership	Group A	*F* = 143.49***	2, 62	0.928 (ns)	3.84*	2.69*	1.16*	0.82
	Group B	*F* = 3.50 (ns)	1.44, 44.60	0.610 (*p* < 0.001)	0.13*	0.03 (ns)	0.09 (ns)	0.1
Self-Efficacy	Group A	*F* = 4.18*	1.64, 50.91	0.782 (*p* = 0.025)	0.06 (ns)	0.16*	0.10 (ns)	0.12
	Group B	F = 66.17***	1.43, 44.17	0.596 (*p* < 0.001)	2.41*	2.13*	0.28 (ns)	0.68

* < 0.05, *** < 0.001, ns = non-significant.

### Qualitative results

**Figure 3:**
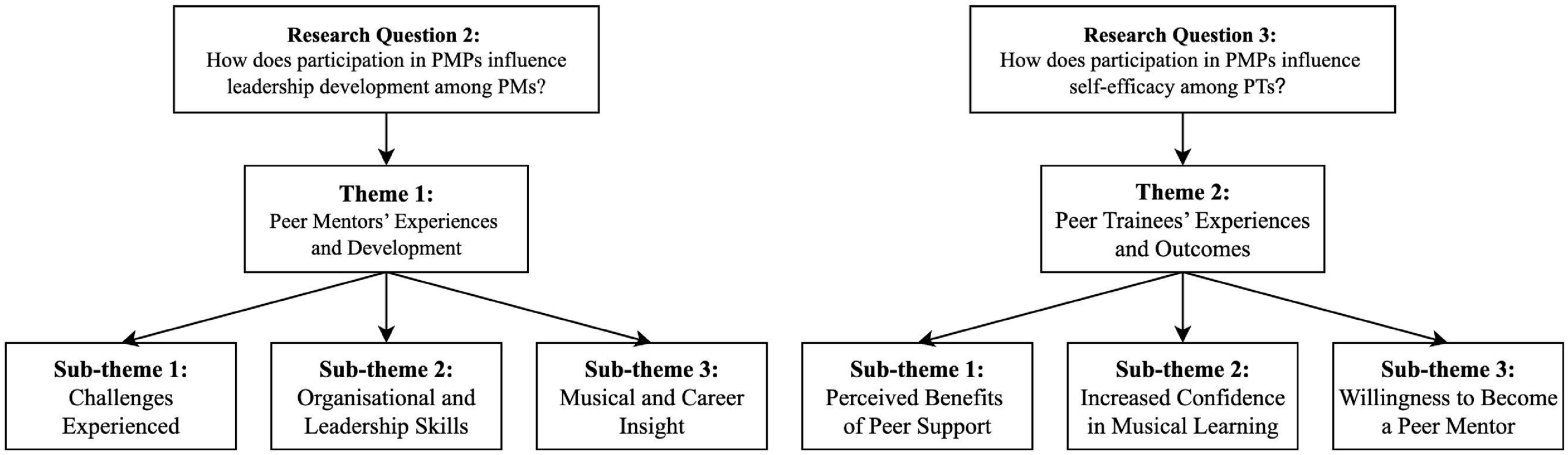
Overview of themes and subthemes. Themes and subthemes were generated through thematic analysis and organized in relation to research questions 2 and 3.

The interviews began with self-introductions, and participants generally engaged naturally and cooperatively. Guided by Braun and Clarke’s (2006, 2019) thematic analysis framework, the interview data were synthesized into two main themes and six subthemes: (1) Peer Mentors’ Experiences and Development; (2) Peer Trainees’ Experiences and Outcomes (see Figure 3).

#### Theme 1. Peer mentors' experiences and development

##### Theme 1.1: challenges experienced

Four primary challenges were identified. Participants C1, C5, and C7 described difficulties managing the dual roles of ‘peer’ and ‘mentor.’ For example, C1 stated, “I wanted to stay close to them, but I also couldn’t lose the authority of being a senior.” C2 and C6 raised concerns regarding their mentoring competence. C2 shared, “Sometimes I worry that my explanations are not clear enough and might confuse them.” C3, C4, C8, and C10 cited time management as the most pressing issue, pointing to the strain of added responsibilities on top of demanding academic schedules. C9 highlighted limited trainee engagement: “I was well prepared, but they didn’t seem involved, which was frustrating.”

##### Theme 1.2: organisational and leadership skills

Most PMs perceived improvement in both organizational and leadership abilities. C2 commented, “I used to do things last minute, but now I make checklists and plan each step. I also feel more confident guiding others.” C1 echoed this view, noting that the program enabled him to “coordinate different paces in the group and keep everyone on track.” In contrast, C3, C5, and C10 expressed more reserved opinions. C5 remarked, “I know how to organize now, but that’s not the same as leading. When they don’t follow my lead, I feel discouraged.” C10 expressed doubts about equating task completion with leadership. C8 identified personal tendencies as a constraint: “I’m not someone who gives orders naturally, so I made progress, but still tend to be passive.” Similarly, C9 reported little personal growth: “I feel the same as before, and didn’t have an ‘aha’ moment.” Despite these varied perspectives, most PMs acknowledged a degree of improvement in coordination and planning skills.

##### Theme 1.3: musical and career insight

Most participants reported acquiring new insights and learning outcomes. C1 and C6 indicated that the mentoring process helped them articulate their individual learning trajectories. C1 explained, “I used to practice without much thought, but now I reflect on why I do things and whether there’s a better way.” C2, C3, and C10 reported an enhanced understanding of music theory, particularly in relation to explaining structure and rhythm. C4 and C8 described increased awareness of rehearsal details. As C4 noted, “To explain clearly, I had to first sort out how each note should be handled.” C5 and C7 engaged in deeper reflection on career development. C7 remarked, “I’ve realized that teaching and communication are skills that may shape my future path.” C9 acknowledged limited personal development: “Maybe because of my lack of experience, I mostly followed the steps without much deep thinking.” Overall, most PMs developed greater self-awareness in both musical learning and career planning, though the extent of impact differed across participants.

#### Theme 2. Peer trainees’ experiences and outcomes

##### Theme 2.1: perceived benefits of peer support

Most students reported experiencing concrete and positive outcomes. T1 and T8 shared that receiving recognition from their PMs significantly boosted their confidence. T1 stated, “My mentor told me that my sense of rhythm had improved a lot. That was the first time I felt my effort was truly acknowledged.” This form of positive feedback was perceived as a key source of emotional support. Meanwhile, T2 and T6 emphasized the value of precise corrective feedback. T6 noted, “I had never noticed my timing issues until he pointed them out. After that, my practice became much smoother.” Some students also emphasized gaining practical playing techniques. T9 shared, “I used to repeat whole pieces, but now I know I can practice slowly in sections and then combine them. It’s much more efficient.” Notably, T4 reported that the interaction with the peer mentor helped clarify their career aspirations. “He told me why he chose music education, and I started thinking whether this could be a path for me too.”

##### Theme 2.2: increased confidence in musical learning

Most PTs reported that participating in the programme substantially improved their self-confidence, particularly in performance and ensemble contexts. T1 shared, “I used to be afraid to play in front of others. Now I voluntarily ask my mentor to listen to my practice, and I feel I have made meaningful progress.” T6 and T8 expressed similar views, noting that they had become more comfortable communicating openly and taking musical risks under their PMs’ encouragement. T3 added, “When I was able to keep up with the ensemble, I truly felt capable.” T4 explained that this growth in self-confidence resulted not only from improved skills but also from a shift in mindset. “I don’t get frustrated anymore if I play a wrong note. My mentor told me ‘this is part of the learning process,’ which helped me feel more at ease.” T5 and T9 emphasized breakthroughs in self-expression. T5 said, “I had never voiced my opinions during rehearsals, but this time, I did.”

##### Theme 2.3: willingness to become a peer mentor

Most PTs expressed a strong desire to become PMs in the future. T1, T2, T4, T5, T8, T9, and T10 indicated they were highly motivated and wished to reciprocate the support they had received. T5 remarked, “If I have the chance, I would like to help new students, just like I was helped.” T8 added, “Seeing how responsible and dedicated my mentor was makes me want to become someone like that too.” However, T3 and T6 expressed reservations regarding their readiness and emphasized the need for structured training before assuming the mentor role. T7 admitted that they were not sufficiently prepared to assume the mentor role, stating, “I feel I’m not prepared enough. Maybe after gaining more experience, I’ll feel more confident.”

## Discussion

The present study examined the potential impact of PMPs in higher music education and found that, within a dual-role structure, participants exhibited role-related developmental trajectories over time. Students serving as PMs demonstrated increased organisational awareness and a clearer sense of guidance, suggesting that leadership behaviors may gradually emerge through sustained task engagement and clearer role identification. Students serving as PTs reported more positive self-evaluations, with gains in self-efficacy arising from sustained interaction and vicarious learning experiences.

These quantitative findings from Phase 1 are best interpreted as time-dependent developmental patterns rather than definitive causal effects. Given the quasi-experimental design and the inherent differences in year level, experience, and role positioning, the analysis focused exclusively on within-group changes, and the repeated-measures ANOVA results should therefore be interpreted with appropriate caution, as they reflect developmental change over time rather than causal or comparative effects. After accounting for potential sources of heterogeneity, the study provides preliminary evidence for the developmental value of PMPs.

Although the increase in self-efficacy among PMs was statistically significant, its effect size was relatively modest. As a core outcome, this pattern is nonetheless educationally meaningful, as it suggests the consolidation of mentoring readiness, leadership-related confidence, and emerging professional role identity rather than dramatic changes in self-belief (Byrne et al., 2022). Likewise, although leadership development among PTs showed a relatively small effect size, this core outcome remains developmentally meaningful, reflecting the gradual and indirect internalization of leadership behaviors through collaborative participation rather than formal role enactment (Gafni Lachter and Ruland, 2018).

The quantitative trends observed in Phase 1 of the study align with findings from recent studies on peer learning and interaction in music education published in 2025. Sun et al. (2025) showed that structured peer interaction in Chinese university music ensembles was associated with improved self-efficacy and social relatedness. Jones and Wendt (2025) observed sustained gains in mentees’ self-efficacy in a higher education peer mentoring intervention, while Zeb et al. (2025) found that interpersonal and feedback-focused interventions strengthened students’ self-efficacy by enhancing perceived support and emotional regulation. These findings are consistent with earlier critiques of traditional master–apprentice pedagogies and underscore the complementary role of structured peer interaction in higher music education (Gaunt and Westerlund, 2013; Hanken, 2016). Gaunt and Westerlund (2013) argue that traditional master–apprentice pedagogies often overlook the potential of peer learning, despite the inherently social nature of music study. The PTs’ improvements in self-efficacy through demonstration, feedback, and collaborative practice support this perspective and underscore the pivotal role of vicarious experiences in music learning. Goodrich (2021b) further demonstrates that mentors’ sharing of experiences can strengthen the learning process, suggesting that the structured design of the PMPs helped stabilize and facilitate peer-learning interactions. Reid and Duke (2015) argue that informal peer interactions vary in autonomy and form depending on instrumental cultures; the present findings extend this view by showing that peer learning requires contextual scaffolding to function effectively and sustainably. Consistent with Guzzetta’s (2019) finding that learners in peer-teaching contexts gradually assume greater levels of agency, the heightened sense of responsibility and engagement observed among participants indicates that peer mentoring may promote not only skill acquisition but also emerging role awareness and learning agency.

Phase 1 results indicate a gradual increase in PMs’ leadership behaviors over time. This pattern addresses a documented gap concerning PMs’ leadership development in music education (Ilchuk et al., 2024). This pattern can be interpreted through Bandura’s (1977) forethought model, which posits that when individuals are assigned explicit responsibilities and expected to influence others, their goal management and guidance tendencies strengthen in response to situational demands. Role expectations and socially interactive tasks provide a structured environment that supports the development of organisational and coordination skills. Interview data indicate that PMs developed more effective communication strategies and a sense of “being needed” as they coordinated rehearsals, delegated tasks, and motivated peers, thereby exhibiting more proactive guidance behaviors in group settings (Sims and Weinberg, 2024). The sustained improvement in leadership behaviors following the intervention further suggests that enacting leadership roles may strengthen the connection between behavior and identity. For example, participant C1’s description of “shifting between being a friend and a guide” illustrates ongoing role calibration during social interaction, aligning with transformational leadership theory, which posits that supporting others’ growth can inspire a greater willingness to lead, particularly in music-learning environments that lack formal authority (Bass and Riggio, 2006). These changes are therefore more plausibly understood as evidence of gradual identity development rather than definitive, causal effects.

The findings also suggest that peer mentoring in music learning should be understood through the lens of socio-emotional development and identity formation. Goodrich’s (2020) research on LGBTQIA+ students shows that peer systems can strengthen emotional stability and identity affirmation by providing safe spaces and social support, particularly in settings that lack formal authority. The shifts observed in PMs’ and PTs’ role awareness, self-efficacy, and sense of belonging align with this socio-emotional pathway, indicating that peer relationships in music-learning environments fulfil not only instructional functions but also shape identity development and behavioral tendencies. Accordingly, the structured tasks and role frameworks of the PMPs may function as a “social mirror,” enabling students to clarify their self-positioning and to develop more stable career expectations and leadership orientations through repeated cycles of collaboration and feedback. This provides new evidence for the socio-psychological dimensions of peer-learning theory in music education.

Phase 1 results show relatively small shifts in PTs’ leadership behavior. However, the modest pre–post improvements suggest that students in non-leadership roles may gradually encounter and begin to internalise leadership behaviors through collaborative engagement (Kritz et al., 2020). Interview data support this pattern: several PTs demonstrated emerging responsibility and organisational tendencies during task distribution and joint practice, indicating that role learning may unfold through participation itself rather than relying entirely on formal positional structures (Gee, 2000; Komives et al., 2005). The increase in musical self-efficacy among Group B is also noteworthy, aligning with mechanisms of vicarious experience and social persuasion. Mentor demonstrations and timely feedback helped PTs form more positive judgements of their abilities and develop a stronger sense of control. Interview accounts from T6 and T1, such as “my direction became clearer” and “I felt seen,” highlight the importance of belonging, recognition, and psychological safety in the development of self-efficacy. These results echo prior findings that supportive peer relationships and positive appraisal enhance students’ perceived control, learning engagement, and emotional stability (Zeb et al., 2025).

### Implication for practice

In Chinese conservatoires, hierarchical culture and master–apprentice traditions remain deeply embedded and continue to shape peer-mentoring dynamics. Existing research in Chinese music education has focused primarily on teacher–student relations or group identity (e.g., Guan et al., 2023; Sun et al., 2025), leaving limited attention to how peers negotiate roles and interactional patterns within such symbolic hierarchies. Within this context, senior students are often granted experiential advantage and symbolic authority, while junior students tend to adopt deferential, upward-learning positions. Although this structure facilitates mentor–mentee relationships, it may also reinforce PTs’ dependence on mentors and constrain opportunities for autonomous exploration. Situated within the Chinese conservatoire context, these findings respond to calls for empirical evidence from non-Western, performance-oriented music education settings (Rector, 2025).

The findings further suggest that music learning involves not only skill development but also gradual identity formation. By clarifying role expectations and providing a relatively low-risk and supportive interactional context, PMPs enable students to receive continuous feedback and role affirmation, thereby supporting both competence and identity development. Given that identity formation is often accompanied by emotional fluctuation and self-doubt (Papageorgi et al., 2007), the more balanced environment fostered by PMPs may be better suited than traditional hierarchical pedagogies to address these developmental needs. Accordingly, the observed changes are best interpreted as evidence of gradual identity development rather than direct causal effects.

### Limitations and future research

This study has several limitations. First, it focused exclusively on within-group changes and did not include between-group comparisons, limiting the ability to draw causal inferences. Future research may adopt more advanced analytical approaches, such as mixed-design ANOVA, ANCOVA, or multilevel modeling, to examine potential group differences or dyadic interdependence. Second, the sample was drawn from a single institution with relatively homogeneous disciplinary backgrounds, which restricts external validity. Future studies should include participants from multiple institutions and artistic disciplines to enhance the generalisability of the findings. Third, although the present study implemented a two-month intervention and included a one-month follow-up assessment, the overall study period primarily captures short-term change and maintenance, making it difficult to evaluate the long-term sustainability of mentoring effects. Future research should extend both the intervention duration and the follow-up period (e.g., 6–12 months) to more comprehensively assess the stability and durability of peer mentoring outcomes. Despite these limitations, the study provides preliminary but meaningful evidence for the value of dual-role peer mentoring structures in arts education.

## Conclusion

This study explored the use of peer mentoring in higher music education and found that the PMPs can be effectively integrated into performance-based learning contexts. The program design, which incorporated emotional support, skill guidance, experience sharing, and performance preparation, was compatible with the practical routines of music training and supported sustained student engagement.

Participation in the program was associated with different forms of developmental change. Students who assumed mentoring responsibilities demonstrated increased organisational awareness and a stronger sense of responsibility through repeated guidance and coordination activities, indicating that leadership-related behaviors can emerge through sustained participation in peer-based learning tasks. At the same time, students receiving peer support showed a clear and lasting improvement in musical self-efficacy. Ongoing feedback, demonstration, and supportive interaction contributed to greater confidence in learning and performance, even though changes in leadership behavior were less pronounced.

Overall, the findings indicate that peer mentoring can complement traditional instruction in higher music education by enhancing learning confidence and collaborative engagement in conservatoire training contexts.

## Data Availability

The raw data supporting the conclusions of this article will be made available by the authors, without undue reservation.
