# Supplementary material for: The impact of peer mentoring on leadership and self-efficacy in higher music education: a mixed-methods study
Source: Front Psychol. 2026 Feb 9;17:1756556. doi: 10.3389/fpsyg.2026.1756556 (PMC12926378; doi:10.3389/fpsyg.2026.1756556)
Supplement: Supplementary file 1 [file Data_Sheet_1.DOCX]

**Appendix A Matching Dimensions and Scoring Criteria**

| Dimension | Description | Scoring Criteria |
| --- | --- | --- |
| Specialization Alignment | Whether the PM and PT are from the same musical specialization (e.g., vocal, piano, instrumental) | 2 points: Same specialization;  1 point: Related specialization (e.g., same keyboard family / same stage-performance category);  0 points: Clearly different specialization |
| Performance/Skill-Area Similarity | Whether their primary performing areas or skill domains are similar | 2 points: Same primary instrument/voice type (e.g., both soprano, both violin);  1 point: Same category but different section/level;  0 points: Substantial differences in performance domain |
| Match Between PM’s Strengths and PT’s Learning Needs | Whether the PM has a clear advantage in the specific area where the PT needs support | 2 points: PM is clearly stronger in the PT’s area of need, and PT explicitly requires support;  1 point: PM has a slight but relevant advantage;  0 points: Advantage is minimal or unrelated to PT’s needs |
| Learning Goal and Interest Compatibility | Whether their learning objectives and developmental goals are aligned | 2 points: Highly aligned goals (e.g., preparing for the same competition or similar postgraduate plans);  1 point: Partially aligned;  0 points: Goals differ significantly or are unclear |
| Scheduling Compatibility | Whether their weekly schedules allow regular meetings for PMP activities | 2 points: Multiple overlapping time slots each week that enable regular sessions;  1 point: Some overlap, but fragmented;  0 points: Little or no overlap, making regular meetings difficult |

**Appendix B Item-Objective Congruence Values for Content Validity of Peer Mentoring Programs**

| Dimension | Expert 1 | Expert 2 | Expert 3 | Expert 4 | Expert 5 | Average |
| --- | --- | --- | --- | --- | --- | --- |
| Emotional Support | 1 | 1 | 0 | 1 | 1 | 0.8 |
| Skill-based Assistance | 1 | 1 | 1 | 1 | 1 | 1 |
| Experience Sharing | 1 | 0 | 1 | 1 | 1 | 0.8 |
| Stage Preparation and Collaboration | 1 | 1 | 1 | 1 | 1 | 1 |

**Appendix C Student Leadership Behavior Scale (SLBS)**

| Taking initiative and modeling the way   1. I take the initiative to speak up and take action. 2. I behave as a role model to members. 3. I always ensure that I follow through on what I have promised members. 4. I seek feedback from others about my performance. 5. I make decisions on my own and take responsibility for that decision even when others contradict me. |
| --- |
| Challenging the process   1. I seek out challenging opportunities that will promote my personal growth. 2. In order to achieve my goals, I try to take up challenges even at the risk of failure. 3. I try to choose the path that will enable personal growth, even if it is difficult. 4. I am always looking for new ways of ensuring that the group presents good results. 5. Whenever I experience setbacks or failures, I think positively and overcome these quickly. |
| Sharing goals   1. I communicate what the group’s goals are to all members in a way that they can understand. 2. I make an effort to ensure that members reach a common understanding. 3. I discuss with members the direction in which the group should aim towards. 4. I motivate members. 5. I present an appealing vision that excites members. |
| Managing goals and tasks   1. I set specific goals that are obtainable through hard work. 2. I make specific plans aimed toward achieving goals, such as deciding members’ roles and task timelines. 3. If disproportionate burden is placed onto any one member, I adjust that person’s role flexiblely. 4. I pay attention to the progress of members’ tasks. 5. I try to predict the future as far as possible and set goals and plans accordingly. |
| Task-oriented support   1. I do the most I can to make it easier for members to progress in their tasks. 2. I create an atmosphere wherein members feel safe expressing their opinions. 3. I help my members to display their strengths. 4. I am aware of whether or not members are having trouble and provide appropriate advice. 5. I provide honest feedback to members in order to improve the team’s overall achievement. |
| People-oriented support   1. I always take members’ feelings into account. 2. I prioritize my members’ interests rather than my own. 3. I treat members with compassion and without unduly reproaching them if and when they fail to produce results. 4. I treat members with trust and respect. 5. I express my gratitude when members do a good job. |

**Appendix D General Musical Self-Efficacy Scale (GMSES)**

| Self-efficacy for music learning   1. I am confident that I can adequately prepare the repertoire for this performance. 2. One of my problems is that I don't start preparing for this particular performance when I should (this is about attitude, not lack of time). 3. If things don’t go well in the first rehearsals, I continue practicing until I am satisfied. 4. When I set important goals related to this performance, I almost never achieve them. 5. I will probably give up preparing for this performance before finishing it completely. 6. When I encounter a difficulty during preparation, I can persist until I overcome it. 7. Once I have decided to perform, I fully concentrate on working on the music. 8. When I play the new program for the performance, I quickly stop if it doesn't go well in the first attempts. 9. The prospect of failing this performance makes me work harder in its preparation. 10. It is likely that I will stop preparing for this performance easily. 11. I am not able to face most of the problems that may arise during preparation. |
| --- |
| Self-Efficacy for Musical Performance   1. I am convinced that I can carry out the performance successfully. 2. I have set high goals for this performance, but I will hardly achieve them. 3. I will probably avoid or modify the most demanding passages in the performance. 4. If I perceive that the context around this performance is very stressful (audience, venue, preparation time, repertoire), I try to avoid it. 5. If something unexpected happens during the performance, I can handle it well. 6. If the program is too difficult for me, I will probably try to avoid the performance. 7. I feel insecure about the interpretation for this performance. 8. If I face a difficulty during the performance, I will probably not be able to overcome it. 9. I am able to overcome the problems that may arise during the performance. 10. The prospect of failing in this performance makes me work harder. 11. I am capable of delivering a good performance. |

**Appendix E Item-Objective Congruence Values for Content Validity of Interview Questions**

| Role | Questions | Expert 1 | Expert 2 | Expert 3 | Expert 4 | Expert 5 | Average |
| --- | --- | --- | --- | --- | --- | --- | --- |
| PMs | Q1 | 1 | 1 | 1 | 1 | 1 | 1 |
|  | Q2 | 1 | 0 | 1 | 1 | 1 | 0.8 |
|  | Q3 | 1 | 1 | 1 | 1 | 0 | 0.8 |
| PTs | Q1 | 1 | 1 | 1 | 1 | 1 | 1 |
|  | Q2 | 1 | 1 | 0 | 1 | 1 | 0.8 |
|  | Q3 | 1 | 1 | 1 | 1 | 1 | 1 |
